# *MYCN*-driven metabolic remodelling of the tumour microenvironment in neuroblastoma: implications for stromal biology and CAF heterogeneity

**DOI:** 10.1007/s10555-026-10355-w

**Published:** 2026-07-24

**Authors:** Mekonnen Sisay Shiferaw, Klaartje Somers, Zaklina Kovacevic

**Affiliations:** 1 Department of Physiology, School of Biomedical Sciences, Faculty of Medicine and Health, University of New South Wales, Sydney, NSW 2052 Australia; 2 School of Pharmacy, College of Medicine and Health Sciences, Wollo University, Dessie, Ethiopia; 3 Children’s Cancer Institute, Minderoo Children’s Comprehensive Cancer Centre, Sydney, NSW Australia; 4 School of Clinical Medicine, University of New South Wales, Sydney, NSW Australia

**Keywords:** Neuroblastoma, *MYCN*, Tumour microenvironment, Cancer-associated fibroblasts, Metabolic reprogramming

## Abstract

Neuroblastoma (NB) is the most common extracranial solid tumour of childhood and remains a leading cause of paediatric cancer mortality, particularly in high-risk disease driven by *MYCN* amplification. Although *MYCN* is a central oncogenic driver, its role as a transcription factor has limited direct therapeutic targeting, shifting attention toward downstream metabolic and microenvironmental dependencies. Increasing evidence indicates that *MYCN*-driven metabolic rewiring extends beyond tumour-intrinsic processes to reshape the tumour microenvironment (TME), influencing immune composition and stromal dynamics. Recent advances in single-cell and spatial profiling technologies have revealed substantial heterogeneity within the NB TME, highlighting complex interactions between tumour cells, immune populations, and stromal components. Among these, cancer-associated fibroblasts (CAFs) have emerged as key regulators of extracellular matrix architecture, immune modulation, and metabolic crosstalk. However, CAF identity, functional diversity, and lineage relationships in NB remain incompletely defined, with significant overlap between tumour-intrinsic mesenchymal programs and stromal fibroblast signatures. In this review, we synthesise current understanding of *MYCN*-driven metabolic reprogramming and its impact on CAF heterogeneity and immune regulation. We integrate insights from adult cancers with emerging data in NB to critically evaluate CAF functional states, including inflammatory and myofibroblastic programs, and their roles in shaping tumour progression, immune exclusion, and therapeutic response. By framing NB as a *MYCN*-remodelled tumour ecosystem, this review identifies key knowledge gaps in stromal biology and highlights the need to resolve CAF heterogeneity and tumour–stroma interactions. These insights have broader implications for *MYC*-driven malignancies and support the development of integrated therapeutic strategies targeting both tumour cells and their supportive microenvironment.

## Introduction

Neuroblastoma (NB), a solid tumour originating from sympathetic neural crest cells, represents the most common extracranial solid malignancy of childhood [1] and accounts for 15% of paediatric cancer-related deaths [2]. NB is a clinically and biologically heterogeneous disease, with variability in tumour biology, genetic alterations, and age at diagnosis. This heterogeneity underpins its stratification into low-, intermediate-, and high-risk groups according to the International Neuroblastoma Risk Group (INRG) or Children’s Oncology Group (COG) classification system [3–6].

Clinical behaviour ranges from spontaneous regression in some low-risk cases to aggressive progression in high-risk disease, which often persists despite intensive multimodal therapy. While patients with low- and intermediate-risk NB generally achieve favourable event-free and overall survival with current treatment strategies [7–9] outcomes for high-risk NB remain poor, highlighting the urgent need for improved therapeutic approaches. Increasing evidence suggests that tumour progression and treatment resistance are shaped not only by intrinsic tumour biology but also by dynamic interactions within the tumour microenvironment (TME). A deeper understanding of the complex crosstalk between NB cells and their surrounding stromal components is therefore critical for the development of more effective treatment strategies.

In this review, we examine the cellular and molecular complexity of NB, with particular emphasis on tumour heterogeneity and *MYCN*-driven remodelling of the TME. We discuss how metabolic rewiring, immune modulation, and stromal interactions collectively shape disease progression and therapeutic resistance. Given the emerging recognition of mesenchymal plasticity in NB and its potential overlap with stromal signatures, we focus specifically on cancer-associated fibroblasts (CAFs), a well-characterized driver of tumour progression in adult malignancies, whose identity, heterogeneity, and function in NB remain incompletely understood. By integrating current evidence and highlighting key knowledge gaps, we aim to define priorities for future investigation into stromal biology in NB.

## NB cellular heterogeneity

NB exhibits marked cellular heterogeneity, with tumour cells broadly classified into two transcriptional states: adrenergic/noradrenergic (ADRN) and mesenchymal (MES) [10]. These states are not fixed lineages but display reversible plasticity. *In vitro*, enforced expression of the mesenchymal transcription factor PRRX1 reprograms ADRN cells towards a MES phenotype, accompanied by widespread transcriptional and super-enhancer remodelling [10]. MES-like tumour cells express markers such as VIM, FN1, SNAI2, and CD44, whereas ADRN cells are characterized by PHOX2A/PHOX2B, DBH, and GATA3-driven regulatory programs. Subsequent studies have confirmed bidirectional transitions between ADRN and MES states in NB cell lines [11].

Functionally, MES states have been associated with therapy resistance and disease progression. MES-like cells demonstrate increased resistance to chemotherapy and are enriched in relapsed tumours [10]. Transition toward MES programs has also been linked to resistance to anti-GD2 immunotherapy in high-risk NB [12]. Computational analyses integrating lineage signatures with bulk transcriptomic datasets further suggest that ADRN-enriched tumours with elevated T-cell inflammation scores are associated with improved survival [13]. However, these correlations are largely based on *in vitro*–derived signatures and computational inference, and functional validation in primary tumour samples remains limited. Moreover, conflicting data exist regarding the prognostic implications of MES *versus* ADRN states, underscoring the complexity of interpreting lineage programs in clinical cohorts [14].

Recent single-cell and single-nucleus transcriptomic studies have challenged the extent to which a bona fide MES tumour state exists *in vivo*. scRNA-seq analyses of primary NB specimens indicate that most malignant cells align along a sympathoadrenal developmental trajectory, with dominant chromaffin-like or adrenergic phenotypes [15, 16]. MES-associated transcriptional programs are detected at low frequency within tumour cells and frequently overlap with stromal populations, including fibroblasts and Schwann cell–derived lineages. Indeed, large-scale integration of multiple single-cell datasets into a unified NB atlas revealed minimal evidence for a distinct *in vivo* MES tumour population, with MES signatures largely mapping to stromal fibroblasts or Schwann-like cells [17]. Similarly, longitudinal multi-omic analyses have suggested that mesenchymal-like programs observed after chemotherapy may partially reflect stromal or fibroblast-associated signatures rather than purely tumour-intrinsic transitions [18].

Collectively, these findings suggest that while ADRN–MES plasticity is well established *in vitro,* its manifestation in primary tumours remains incompletely resolved. Importantly, the transcriptional overlap between MES tumour signatures and stromal fibroblast programs complicates interpretation of bulk and single-cell datasets. Distinguishing tumour-intrinsic MES states from bona fide stromal populations is therefore critical for accurately defining NB heterogeneity and for understanding how the tumour microenvironment contributes to disease progression and therapeutic resistance.

## Overview of the NB microenvironment

The NB TME comprises a dynamic network of immune and stromal cell populations that collectively influence tumour progression and therapeutic response. Immune components include NK cells, T and B lymphocytes, dendritic cells, neutrophils, tumour-associated macrophages (TAMs) and myeloid derived suppressor cells (MDSCs), each capable of exerting context-dependent pro- or anti-tumourigenic effects [19, 20]. In parallel, mesenchymal stromal cells (MSCs) and cancer associated fibroblasts (CAFs) contribute structural support and secrete cytokines, chemokines and extracellular matrix (ECM) components that modulate immune recruitment, tumour cell survival, and metastatic dissemination [21].

Emerging spatial analyses indicate that NB tumours exhibit compartmentalized architecture, with immune cells frequently localized adjacent to mesenchymal populations [15], suggesting active stromal–immune crosstalk. However, despite identification of these cellular constituents, the extent of their heterogeneity and functional interactions within the NB TME remains incompletely defined. In contrast to adult solid tumours, where CAF diversity and immunomodulatory roles are well characterized, stromal biology in NB has only recently begun to receive attention.

Given the marked cellular heterogeneity of NB and its association with therapy resistance, a comprehensive understanding of tumour–microenvironment interactions, including stromal contributions, may be critical for improving outcomes in high-risk disease [22, 23].

### The role of *MYC* in the TME

#### Overview of *MYC* in other cancers

The *MYC* oncogene family comprises three major members—*MYCN, MYCC*, and *MYCL*, encoding the transcription factors N-Myc, c-Myc, and L-Myc, respectively [24–26]. These proteins function as master transcriptional regulators, controlling expression of a substantial fraction of the genome and orchestrating programs governing proliferation, metabolism and cellular growth.

Beyond tumour-intrinsic effects, MYC proteins actively shape the TME. In multiple solid cancers, MYC signalling promotes angiogenesis, modulates CAF activation, and drives immune evasion [27]. For example, in breast cancer, c-MYC regulates tumour-stroma crosstalk, enhancing immune suppression and facilitating tumour invasion [28]. MYC-amplified tumour cells have also been shown to secrete exosomal mediators, such as miR-105, that reprogram neighbouring fibroblasts and alter metabolic support within the TME [29, 30]. These observations position MYC family members not only as drivers of tumour cell proliferation, but also as architects of the broader tumour ecosystem.

#### The impact of* MYCN* in NB tumourigenesis and progression

Genetic aberrations in several proto-oncogenes or tumour suppressor genes may trigger neural crest-derived cells to develop into NB, arresting their differentiation into sympathetic ganglia and chromaffin cells of the adrenal medulla [3, 31]. Among these alterations, *MYCN* amplification represents the most extensively characterized oncogenic driver, occurring in approximately 40–50% of high-risk cases [32–34]. Since its initial identification in the 1980 s [35, 36], *MYCN* amplification has remained a cornerstone prognostic marker associated with aggressive disease and poor outcome in NB [32, 37]. *MYCN*, located on chromosome 2p24, encodes a transcription factor that regulates genes governing proliferation, ribosome biogenesis, protein translation, and metabolism [38]. The oncogenic capacity of *MYCN* is further underscored by the TH-*MYCN* transgenic mouse model, in which enforced *MYCN* expression is sufficient to drive tumours closely resembling human NB [39, 40]. However, NB heterogeneity is not explained by *MYCN* amplification alone, as high-risk and aggressive tumours also harbour recurrent kinase alterations, telomere-maintenance mechanisms, and segmental chromosomal aberrations, including *ALK* activation, *TERT* rearrangement, *ATRX* alteration, 11q deletion, 17q gain, and 1p deletion [41–51]. These recurrent alterations and their relevance to NB heterogeneity are summarized in Table 1.

**Table 1:** Recurrent genomic alterations contributing to neuroblastoma heterogeneity

Genetic alterations	Frequency	Clinical and molecular context	Relevance to NB heterogeneity	References
*MYCN* amplification	All NB: ~ 18–20%; high-risk NB: ~ 40–50%	Most established adverse genomic marker; enriched in aggressive/metastatic high-risk disease	*MYCN* amplification explains a large fraction of high-risk NB	[34, 44, 46]
*ALK* activation (mutation ± amplification)	*ALK* mutation: ~ 10–15% of primary tumours; Pugh et al. reported *ALK* mutation in 9.2% of high-risk cases. *ALK* amplification is less frequent and should be reported separately if used	Occurs across stages; enriched in familial, high-risk and relapsed disease; may cooperate with *MYCN*	Most recurrent actionable kinase lesion in NB; important as it provides a genetically targetable subgroup	[41, 43, 44, 50]
*TERT* rearrangement/telomerase activation	High-risk NB: ~ 20–25%; Peifer et al*.* reported 23% in high-risk/stage III–IV tumours	Usually high-risk and commonly non-*MYCN*-amplified; one of the major telomere-maintenance mechanisms	Major contributor to aggressive non-*MYCN*-amplified NB; supports the concept that telomere biology is central to high-risk heterogeneity	[42, 45, 46]
*ATRX* alteration/*ALT* phenotype	High-risk NB: up to ~ 8–10%; Pugh et al. reported *ATRX* mutation in 2.5% plus focal deletions in 7.1% of NB; van Gerven et al. reports *ATRX* mutated in up to 8.6% of high-risk NB	Enriched in older children, adolescents and young adults; usually non-*MYCN*-amplified; linked to *ALT*	Defines a biologically distinct telomere-maintenance subgroup that is largely separate from *MYCN*-amplified disease	[43, 46, 47]
11q deletion	High-risk NB: commonly reported in ~ 35–45% of tumours, but exact frequency depends on cohort and assay	Common in aggressive non-*MYCN*- amplified tumours; associated with segmental chromosomal alterations and relapse risk	Major adverse chromosomal subgroup; helps explain high-risk NB heterogeneity outside the *MYCN*-amplified class	[48, 52, 53]
17q gain	Most frequent segmental chromosomal alteration; often reported in > 50% of NB cases, but frequency varies by cohort and detection method	Often co-occurs with *MYCN* amplification, 1p loss, 11q loss or other segmental copy-number lesions	Shows that NB heterogeneity is dominated by chromosomal imbalance and structural/copy-number alterations rather than only recurrent point mutations	[48, 49]
1p deletion	Recurrent adverse segmental loss; reported frequency is cohort/risk dependent	Frequently co-occurs with *MYCN* amplification and aggressive disease features	Considered as a cooperating adverse copy-number lesion rather than an isolated dominant driver	[48, 53]
Predisposition genes (*ALK*, *PHOX2B*)	Familial NB is rare (~ 1–2% of cases). Germline *ALK* explains many hereditary families; *PHOX2B* is a rare hereditary predisposition gene	Hereditary or suspected predisposition NB; relevant when there is family history, multifocal disease or syndromic features	Rare overall but clinically important for genetic counselling, surveillance and familial risk assessment	[41, 54, 55]

Abbreviations: *ALK*, Anaplastic lymphoma kinase; *ALT*, Alternative lengthening of telomeres; *ATRX*, Alpha-thalassaemia/mental retardation syndrome X-linked; *NB*, Neuroblastoma; *PHOX2B*, Paired-like homeobox 2B; *TERT*, Telomerase reverse transcriptase; *1p*, Short arm of chromosome 1; *11q*, Long arm of chromosome 11; *17q*, Long arm of chromosome 17

Despite its central role in NB biology, MYCN is widely regarded as a challenging therapeutic target due to its function as a transcription factor [56]. Consequently, indirect strategies aimed at destabilizing N-Myc or disrupting its downstream programs are under active investigation. These include targeting epigenetic regulators (e.g., PRMT5), interfering with MYCN/MAX interactions, and destabilizing N-Myc protein through inhibition or degradation of Aurora-A kinase (AURKA) [57–60].

Importantly, *MYCN* exerts many of its oncogenic effects through downstream metabolic and signalling pathways. For example, *MYCN* directly upregulates ornithine decarboxylase 1 (ODC1), the rate-limiting enzyme in polyamine biosynthesis [61] and modulates regulators of p53 stability such as MDM2 [62, 63]. These tumour-intrinsic programs extend beyond proliferation to encompass metabolic rewiring and modulation of the surrounding immune microenvironment; themes explored in the following sections.

##### *MYCN* and metabolic reprogramming

Metabolic reprogramming is a hallmark of oncogenesis, enabling tumour cells to meet the heightened bioenergetic, biosynthetic, and redox demands associated with rapid proliferation. In NB, *MYCN* amplification functions not only as a transcriptional driver of cell-cycle progression and growth signalling, but also as a central regulator of metabolic circuitry. Integrative genomic and transcriptomic analyses have demonstrated that *MYCN* profoundly reshapes metabolic gene expression programs in NB [64] with dysregulated pathways including glycerolipid, arginine, and amino acid metabolism strongly associated with high-risk disease [65].

Targeted metabolomic profiling further supports the existence of distinct metabolic landscapes in *MYCN*-amplified and non-amplified tumours [66]. In parallel, machine learning–based analyses have identified metabolic reprogramming-associated prognostic signatures that stratify NB patients according to metabolic activity and clinical outcome [67]. Together, these findings reinforce the concept that *MYCN*-driven metabolic rewiring is not merely a downstream consequence of proliferation, but a defining feature of aggressive disease biology.

In the following sections, we examine key metabolic pathways regulated by *MYCN* in NB and discuss how these tumour-intrinsic adaptations may also influence the surrounding TME including immune cells and CAFs.

A. Polyamine metabolism

Polyamines (i.e., putrescine, spermidine, and spermine) are essential regulators of cell proliferation, nucleic acid stability, and protein synthesis [68, 69]. In cancer, elevated polyamine levels support rapid tumour growth and, importantly, can exert immunosuppressive effects within the TME, thereby facilitating immune evasion and disease progression [70].

In NB, polyamine metabolism is tightly coupled to *MYCN* amplification. *MYCN* co-ordinately regulates both polyamine biosynthesis and uptake by transcriptionally activating key pathway components, including the rate-limiting enzyme ODC1 and transport-associated genes such as SLC3A2 [71, 72]. ODC1 is a direct *MYCN* target, with MYCN/MAX complexes binding E-box motifs in the *ODC1* regulatory region, resulting in increased transcription and enzyme activity, particularly in *MYCN*-amplified tumours and cell lines [61]. This dual control of synthesis and uptake established polyamine metabolism as a central downstream effector of *MYCN*-driven oncogenesis.

Recent work has further refined our understanding of polyamine transport in NB. While SLC3A2 was initially implicated in polyamine import, ATP13A3 has now been identified as the primary mediator of both basal and difluoromethylornithine (DFMO)-induced compensatory polyamine uptake, positioning it as a key regulator of polyamine homeostasis in NB cells [73]. These findings reinforce the concept that *MYCN*-amplified tumours are highly dependent on maintaining intracellular polyamine pools.

Polyamine metabolism may also intersect with epigenetic, metabolic and cell-fate regulation [74]. N-acylspermidines, including succinylated and glutarylated spermidine species, were recently identified as conserved metabolites linked to mitochondrial sirtuin activity, particularly SIRT5, suggesting a potential connection between polyamine derivatives and NAD⁺-dependent deacylation networks [75]. This may be relevant in NB, where sirtuins regulate stress responses, proliferation and differentiation: SIRT5 overexpression protects SH-EP NB cells from oxidative stress and apoptosis [76], SIRT6 inhibition or knockdown induces neurite extension and cell-cycle arrest in BE(2)-C cells [77], and SIRT2 has been reported to act as a histone delactylase that suppresses NB cell proliferation and migration [78]. Thus, MYCN-driven polyamine metabolism may converge with sirtuin-regulated metabolic, chromatin and cell-fate programs, although direct evidence linking acylspermidines to sirtuin activity in NB remains lacking.

Therapeutically, this dependency has been exploited using polyamine-blocking strategies. Inhibition of ODC1 with DFMO, particularly in combination with the polyamine transport inhibitor AMXT 1501, significantly delayed tumour development and extended survival in NB-prone mouse models [72]. In the immunocompetent TH-MYCN model which develops tumours closely resembling NB [79–81], dietary restriction of arginine and proline reduced ornithine levels and synergized with DFMO to further deplete tumour polyamines [82], underscoring the metabolic plasticity of this pathway.

Beyond tumour-intrinsic effects, polyamine metabolism has important immunological consequences. In melanoma and mammary carcinoma models, polyamine-blocking therapy enhanced anti-tumour immunity and synergized with immune checkpoint inhibition [83, 84], suggesting that targeting polyamine metabolism may remodel the TME in addition to impairing tumour growth. Moreover, in pancreatic cancer, CAF-derived acetate was shown to modulate tumour polyamine metabolism *via* the ACSS2–SP1–SAT1 axis [85], highlighting the potential for stromal–tumour metabolic crosstalk through polyamine pathways.

Although direct evidence linking polyamine metabolism to CAF heterogeneity and function in NB remains limited, the strong *MYCN* dependency of this pathway, combined with its established immunomodulatory and stromal interactions in other tumour types, suggests that polyamine metabolism may represent a key interface between tumour-intrinsic metabolic rewiring and microenvironmental remodelling in NB.

B. NAMPT-NAD pathway

Nicotinamide phosphoribosyltransferase (NAMPT) is the rate-limiting enzyme of the nicotinamide adenine dinucleotide (NAD) salvage pathway, catalysing the production of nicotinamide mononucleotide (NMN) from nicotinamide (NAM) and thereby sustaining intracellular NAD pools. NAD is essential for redox balance, ATP production, DNA repair, and the activity of NAD-dependent enzymes such as sirtuins and PARPs. Given the heightened metabolic and replicative demands of cancer cells, many tumours overexpress NAMPT to maintain adequate NAD supply [86].

Although a direct mechanistic link between *MYCN* and the NAMPT–NAD axis has not yet been established in NB, *MYCN*-amplified malignancies appear particularly sensitive to perturbations in NAD metabolism. In *MYCN*-amplified gliomas and other neuroendocrine tumours, increased reliance on glycolysis and metabolic flux renders tumour cells vulnerable to NAMPT inhibition [87–89]. These observations suggest that *MYCN*-driven metabolic rewiring may create a broader dependency on NAD salvage pathways, potentially extending to NB.

Consistent with this concept, pharmacological targeting of NAMPT has demonstrated preclinical efficacy in NB models. Early-generation inhibitors such as FK866 and GMX1777 (CHS828) effectively suppressed NAD synthesis and impaired tumour growth *in vitro* and *in vivo* [90, 91]. More recently, the novel NAMPT inhibitor OT-82 has been shown to induce tumour regression and enhance DNA damage in orthotopic and patient-derived xenograft models of NB [92]. Collectively, these findings support NAD salvage as a metabolic vulnerability in NB, although much of the emerging evidence remains to be validated in peer-reviewed studies.

Beyond its intracellular enzymatic role, NAMPT also exists in an extracellular form (eNAMPT, also known as visfatin), which functions as a cytokine-like mediator within the TME [93, 94]. eNAMPT has been implicated in promoting immunosuppressive signalling and expansion of suppressive immune subsets in several cancer types [95]. In colorectal cancer, eNAMPT activates JAK2–STAT3 signalling in CAFs, driving a pro-metastatic phenotype [96] and pharmacologic NAMPT inhibition attenuates CAF activation, inflammatory cytokine secretion, and stemness-associated programs [97].

Taken together, these findings raise the possibility that NAMPT inhibition in NB may exert dual effects: disrupting tumour-intrinsic NAD metabolism while simultaneously modulating stromal and immune compartments. However, the specific role of the NAMPT–NAD pathway in shaping CAF heterogeneity, immune suppression, or metabolic crosstalk within the NB microenvironment remains largely unexplored and warrants further investigation.

C. Arginine metabolism

Arginine metabolism represents another metabolic vulnerability in NB. Several malignancies exhibit extracellular arginine dependency (auxotrophy) due to reduced expression of urea cycle enzymes required for endogenous arginine regeneration [98]. NB has been shown to display arginine auxotrophy [99, 100], rendering tumour cells dependent on extracellular arginine uptake.

Therapeutically, this vulnerability has been exploited through both inhibition of arginine transport and enzymatic arginine depletion. Blocking cationic amino acid transporter 1 (CAT-1)-mediated arginine uptake *in vitro*, or systemic arginine depletion using recombinant arginase (BCT-100), significantly delayed tumour progression and prolonged survival in murine NB models [101]. Several recombinant arginases, including BCT-100, are currently under clinical investigation in solid tumours, underscoring translational interest in this strategy.

Beyond tumour-intrinsic metabolism, arginine availability has profound immunological implications. Arginine depletion is well known to suppress T cell proliferation and effector function, while altered arginine metabolism can skew myeloid cell activity. Thus, therapeutic arginine depletion may have complex and context-dependent effects within the TME. In contrast, the role of arginine metabolism in CAFs remains poorly defined in NB, representing an underexplored dimension of amino acid competition and metabolic crosstalk in the NB TME.

D. Glucose, lipid and mitochondrial metabolism

Glucose metabolism is central to *MYCN*-driven metabolic reprogramming in NB. *MYCN* amplification is associated with increased expression of hypoxia-inducible factor 1α (HIF-1α), and together these factors promote aerobic glycolysis through transcriptional activation of glycolytic genes [102–104]. This Warburg-like phenotype enhances glucose uptake and lactate production, sustaining rapid proliferation while reshaping the metabolic landscape of the TME. Increased lactate production contributes to TME remodelling as tumour-derived lactate can promote extracellular acidification, impair anti-tumour immune cell function and support immunosuppressive metabolic crosstalk [105]. Consistent with this, elevated lactylation and lactate-metabolism activity in NB have recently been associated with poor prognosis, reduced immune infiltration and impaired NK-cell-mediated cytotoxicity [106].

A key *MYCN*-regulated metabolic effector is SLC16A1, which encodes the lactate transporter MCT1. Pharmacologic inhibition of MCT1 using SR13800 disrupts the NADH/NAD⁺ ratio and reduces intracellular glutathione levels in NB cells, highlighting the importance of lactate export in maintaining redox balance [107]. Unbiased metabolic drug screening further demonstrated that *MYCN*-amplified NB cells are hypersensitive to combinations including MCT1 inhibitors [108], reinforcing glycolysis and lactate transport as actionable vulnerabilities.

Beyond glycolysis, *MYCN*-amplified NB exhibits enhanced oxidative metabolism. Elevated expression of dihydrolipoamide S-succinyltransferase (DLST), a core component of the α-ketoglutarate dehydrogenase complex, is associated with aggressive disease and poor prognosis [109, 110]. DLST facilitates oxidative decarboxylation of α-ketoglutarate to succinyl-CoA, generating NADH for oxidative phosphorylation (OXPHOS), and thereby linking glutamine-derived carbon to mitochondrial respiration. Succinate dehydrogenase (SDH), or mitochondrial respiratory chain complex II, similarly connects the TCA cycle to OXPHOS by oxidising succinate to fumarate and transferring electrons to the electron transport chain. SDHB, which encodes the iron–sulfur subunit of SDH, maps to chromosome 1p36, a region frequently affected by loss of heterozygosity in NB. Although this has prompted investigation of SDHB as a potential target of 1p loss, primary NB analyses did not identify recurrent germline or somatic SDHB mutations, and partial promoter methylation was not associated with SDHB silencing or reduced SDH activity [111]. Thus, while SDH loss can increase succinate and succinate/α-ketoglutarate ratios with potential effects on α-ketoglutarate-dependent dioxygenases, current evidence suggests that SDHB inactivation is unlikely to be a major recurrent mechanism of metabolic rewiring in sporadic NB [112].

The generation of succinyl-CoA may also provide substrate for lysine succinylation, a metabolically sensitive acyl-lysine modification that regulates chromatin accessibility, transcription, DNA damage responses, RNA metabolism and mitochondrial enzyme activity in other cancer contexts [113]. These functions are potentially relevant to *MYCN*-amplified NB, where tumour maintenance depends on *MYCN*-driven transcriptional amplification, ribosome/RNA-processing programs and mitochondrial metabolism. Notably, lysine acetyltransferase 2 A (KAT2A) has recently been implicated as a *MYCN*-associated chromatin regulator in NB [114]. Studies in other systems suggest that KAT2A can use α-ketoglutarate dehydrogenase-derived succinyl-CoA to catalyse histone succinylation [115]. Thus, the DLST–succinyl-CoA–KAT2A axis may represent a plausible but as yet untested mechanism linking mitochondrial metabolism to *MYCN*-dependent chromatin regulation in NB. However, direct evidence linking succinyl-CoA to lysine succinylation in NB remains limited.

Fatty acid metabolism constitutes an additional layer of *MYCN*-driven metabolic adaptation. *MYCN* amplification promotes fatty acid uptake and biosynthesis, leading to glycerolipid accumulation and enhanced tumour survival [116]. Inhibition of β-oxidation selectively reduced viability of *MYCN*-amplified NB cells and decreased tumour burden *in vivo*, whereas non-amplified tumours were less affected [117], indicating a context-specific dependency.

A recent study further demonstrated that *MYCN*-amplified NB cells have a functional dependence on acetate metabolism. Acetate is a short-chain fatty acid metabolite that is converted to acetyl-coenzyme A (acetyl-CoA) by the acyl-CoA synthetase short-chain family member 2 (ACSS2). ACSS2 was found to be elevated in *MYCN*-amplified NB cells, being associated with poor clinical outcomes. Mechanistically, ACSS2-medated acetate metabolism promoted proliferation and tumourigenesis of *MYCN*-amplified NB cells. Importantly, this effect extended beyond metabolism alone — with epigenetic changes also evident. Indeed, acetyl-CoA is the sole substrate for histone acetylation, serving as an epigenetic modulator which was recently demonstrated to directly promote MYCN expression in *MYCN*-amplified NB cells [118].

Epigenetic regulation further integrates *MYCN* signaling with lipid metabolism. MYCN suppresses ELOVL2, a rate-limiting enzyme in docosahexaenoic acid (DHA) biosynthesis, *via* recruitment of polycomb repressive complex 1 (PRC1) and deposition of H2AK119ub, leading to transcriptional silencing [119]. Higher ELOVL2 expression correlates with favourable tumour biology and improved survival, suggesting that *MYCN*-driven lipid rewiring contributes to aggressive disease phenotypes. Conversely, elevated expression of ELOVL6 has been associated with poor prognosis and an immunosuppressive TME in NB cohorts, implicating lipid metabolism in shaping immune contexture [120].

E. Serine-glycine one carbon metabolic pathways

*MYCN*-amplified NB is profoundly dependent on the serine–glycine–one-carbon (SGOC) metabolic network, which supports nucleotide biosynthesis and redox homeostasis. Mechanistically, MYCN cooperates with ATF4 to transcriptionally activate multiple SGOC enzymes, including phosphoglycerate dehydrogenase (PHGDH), the rate-limiting enzyme in *de novo* serine synthesis, as well as key components of the folate-cycle [121]. This transcriptional program channels glycolytic intermediates into serine and glycine biosynthesis, thereby sustaining rapid proliferation and maintaining NADPH production for oxidative stress management.

Consistent with this, PHGDH is strongly expressed in *MYCN*-amplified tumours [100] and functional studies have demonstrated a metabolic dependence on serine biosynthesis in this context. Pharmacologic inhibition of PHGDH significantly reduces tumour growth in MYCN-driven xenograft models, highlighting a targetable vulnerability [122]. Beyond serine synthesis, MYCN directly regulates glycone decarboxylase (GLDC), which catalyses the rate-limiting step in glycine cleavage and generates 5,10-methylenetetrahydrofolate, a key one-carbon donor for nucleotide production [123]. Accordingly, GLDC expression is markedly elevated in MYCN-amplified tumours and cell lines.

MYCN-driven activation of SGOC metabolism may also have epigenetic consequences, as this pathway fuels the folate–methionine cycle and production of S-adenosylmethionine, the universal methyl donor for DNA, RNA and histone methylation [124, 125]). This is particularly relevant in *MYCN*-amplified NB, where *MYCN* cooperates with chromatin regulators including WDR5 and G9a/EHMT2 to sustain activating histone marks at *MYC* target genes and repress neuronal differentiation programs [126].

Importantly, SGOC flux in NB is sustained by integration with other metabolic pathways. Glycolysis provides 3-phosphoglycerate for serine synthesis, while glutaminolysis supports both serine/glycine production and replenishment of the tricarboxylic acid (TCA) cycle via α-ketoglutarate (α-KG) [127]. This coordinated metabolic rewiring renders *MYCN*-driven tumours highly reliant on SGOC pathway activity. Indeed, *MYCN*-induced dependency on SGOC metabolism represents a selective therapeutic vulnerability, as demonstrated by genetic and pharmacologic targeting approaches [104, 128, 129].

While SGOC pathway dependencies have been best characterized in *MYCN*-driven NB cells, emerging evidence from immunometabolism and fibroblast biology indicates that serine/one-carbon flux can regulate T cell programs, macrophage inflammatory states, and collagen synthesis in activated fibroblasts [130–132], raising the possibility that tumour SGOC rewiring may also shape the NB microenvironment.

F. Glutamine metabolism

Glutamine metabolism represents another central metabolic dependency in *MYCN*-amplified NB. *MYCN* not only enhances glutamine uptake but also drives glutaminolysis to sustain TCA cycle anaplerosis, converting glutamine to α-KG to support bioenergetic and biosynthetic demands [133, 134]. Indeed, *MYCN*-amplified tumours exhibit elevated *de novo* glutamine synthesis and heightened reliance on glutamine flux [117]. This metabolic rewiring ensures sustained ATP production, redox homeostasis, and provision of intermediates for nucleotide and amino acid biosynthesis. Consistent with this, enhanced glutamine transport has been shown to couple mitochondrial glutamine utilization to ATP generation and glutathione synthesis in pancreatic cancer cells, reinforcing the importance of glutamine in supporting oxidative stress tolerance [135].

Beyond tumour-intrinsic metabolism, glutamine availability has important implications for the TME, particularly CAFs. Glutamine-derived carbon and nitrogen contribute to proline synthesis, a critical precursor for collagen production. A subset of CAFs characterized by high COL11A1 expression modulates ECM stiffness and promotes tumour growth through metabolic and pro-survival signalling pathways [136–139]. In NB co-culture systems, silencing of COL11A1 in CAFs attenuated tumour invasion, underscoring the functional relevance of stromal collagen programs [140]. Supporting this link between glutamine metabolism and matrix production, pyrroline-5-carboxylate reductase 1 (PYCR1), a key enzyme in proline synthesis from glutamine, is highly expressed in CAFs in several tumour types [141]. Moreover, aldehyde dehydrogenase family 18 member A1 (ALDH18A1), which catalyzes the rate-limiting step in proline and collagen synthesis, forms a positive feedback loop with MYCN, as ALDH18A1 regulates MYCN expression and is reciprocally transactivated by MYCN [142]. Hypoxia, a common feature of the NB TME, further induces ALDH18A1 expression, potentially amplifying this axis [143].

These findings suggest that *MYCN*-driven glutamine metabolism extends beyond tumour-cell anaplerosis to influence ECM remodelling and stromal activation. By fuelling proline and collagen synthesis, glutamine metabolism may reinforce a mechanically and metabolically supportive niche for tumour progression. However, while *MYCN* amplification clearly establishes tumour-intrinsic glutamine dependence, the extent to which *MYCN*-driven glutamine flux directly reprograms CAF metabolism in NB remains poorly defined and represents an important area for future investigation.

Collectively, these studies reveal that *MYCN* amplification orchestrates a coordinated metabolic program rather than isolated pathway activation. *MYCN*-driven tumours exhibit heightened dependence on polyamine biosynthesis and uptake, NAD salvage, glutamine anaplerosis, arginine availability, aerobic glycolysis, and fatty acid remodelling. These adaptations sustain rapid proliferation by maintaining nucleotide synthesis, mitochondrial respiration, redox balance, and membrane biosynthesis. Notably, many of these pathways converge on metabolite recycling and salvage mechanisms such as polyamine uptake, NAD regeneration, and amino acid auxotrophy, suggesting that *MYCN*-amplified NB operates in a state of sustained metabolic flux that necessitates continuous nutrient replenishment and redox buffering.

Furthermore, *MYCN*-driven metabolic rewiring may influence NB biology not only by sustaining bioenergetic, biosynthetic and redox demands, but also by altering metabolite pools that interface with chromatin regulation. Several metabolites generated or consumed through MYCN-associated pathways, including acetyl-CoA, succinyl-CoA, α-ketoglutarate, S-adenosylmethionine and NAD⁺, function as substrates or cofactors for chromatin-modifying enzymes [144, 145]. These metabolite-sensitive mechanisms may provide a means by which *MYCN* reinforces oncogenic transcriptional programs and differentiation blockade, although their direct contribution to epigenome remodelling in NB remains incompletely defined.

Importantly, these metabolic dependencies extend beyond tumour-intrinsic bioenergetics. Enhanced lactate export altered amino acid availability, polyamine accumulation, and lipid remodelling have the potential to reshape the TME by influencing immune cell function, stromal activation, and ECM composition. Although emerging evidence indicates that *MYCN*-driven metabolic outputs can modulate immune and stromal compartments, the precise mechanisms through which *MYCN*-amplified tumour cells metabolically reprogram CAFs and other microenvironmental constituents remain incompletely defined. Defining these tumour–stroma metabolic interfaces represents a critical next step for understanding NB progression and for identifying combinatorial therapeutic strategies that target both tumour metabolism and the supportive niche. Given the central role of immune dysfunction in NB progression and therapeutic resistance, understanding how *MYCN*-driven metabolic rewiring intersects with immune regulation is critical.

##### *MYCN* and NB immune microenvironment

Multiple studies consistently report that *MYCN*-amplified NBs exhibit reduced immune infiltration compared with non-amplified tumours, including diminished CD4⁺ and CD8⁺ T-cell and NK-cell content [146–148]. Bulk transcriptomic analyses coupled with computational immune deconvolution have demonstrated an inverse correlation between *MYCN* amplification and leukocyte infiltration, with higher cytotoxic T-cell signatures associated with improved survival, particularly in non-*MYCN* amplified tumours [146, 147]. Immunohistochemical validation in several cohorts supports these observations, reinforcing the concept that *MYCN* amplified tumours are relatively immune cell-depleted.

Beyond reduced lymphocyte abundance, *MYCN* appears to influence immune recognition mechanisms. In NB cell lines, MYCN expression inversely correlates with surface expression of ligands for NK-cell activating receptors, including NKG2D (MICA/B, ULBP1–3) and DNAM-1 (PVR/CD155, Nectin-2), findings partially validated in primary tumour specimens [149]. Additionally, transcriptomic analyses suggest that *MYCN* amplification is associated with a Th1-to-Th2 shift and macrophage polarization toward an M2-like phenotype [150] consistent with an immunosuppressive TME.

Single-cell approaches have begun to refine this picture. Cross-species scRNA-sequencing analyses of TH-MYCN-driven mouse models and human NB specimens revealed low T-cell content, heterogeneous macrophage populations, and signatures consistent with myeloid-derived suppressor cells [151]. Similarly, scRNA-seq profiling of human NB tumours has identified diverse immune cell states, including multiple myeloid and lymphoid subsets [152]. However, stratification by *MYCN* amplification status has been limited in these studies, leaving unresolved how MYCN specifically shapes immune heterogeneity at single-cell resolution.

An additional layer of complexity arises from the presence of cMYC-driven NBs, which account for a subset of high-risk tumours [153, 154]. Given the shared biology of MYC family transcription factors and evidence that oncogenic MYC can directly promote immune evasion—through induction of checkpoint molecules and suppression of antigen presentation pathways [155, 156] — it is plausible that cMYC-driven NB may engage similar immunosuppressive mechanisms. However, systematic analyses distinguishing NMYC — from cMYC-driven immune landscapes remain limited.

Despite the emerging consensus that *MYCN*-amplified tumours are relatively immune-poor, important limitations remain. Much of the existing evidence derives from bulk RNA-seq datasets with computational immune deconvolution, which infer rather than directly measure immune subset abundance and lack spatial resolution. Conventional IHC validation, while informative, does not capture high-dimensional immune phenotypes or cell–cell interactions within intact tissue architecture. Furthermore, many studies do not clearly distinguish *MYCN* amplification from elevated MYCN expression, complicating interpretation of causality. Recent paediatric tumour-immune microenvironment analyses have also demonstrated weak concordance between computational deconvolution and histological assessment of T-cell infiltration [157] underscoring the need for integrated single-cell and spatially resolved approaches.

**Fig. 1:**
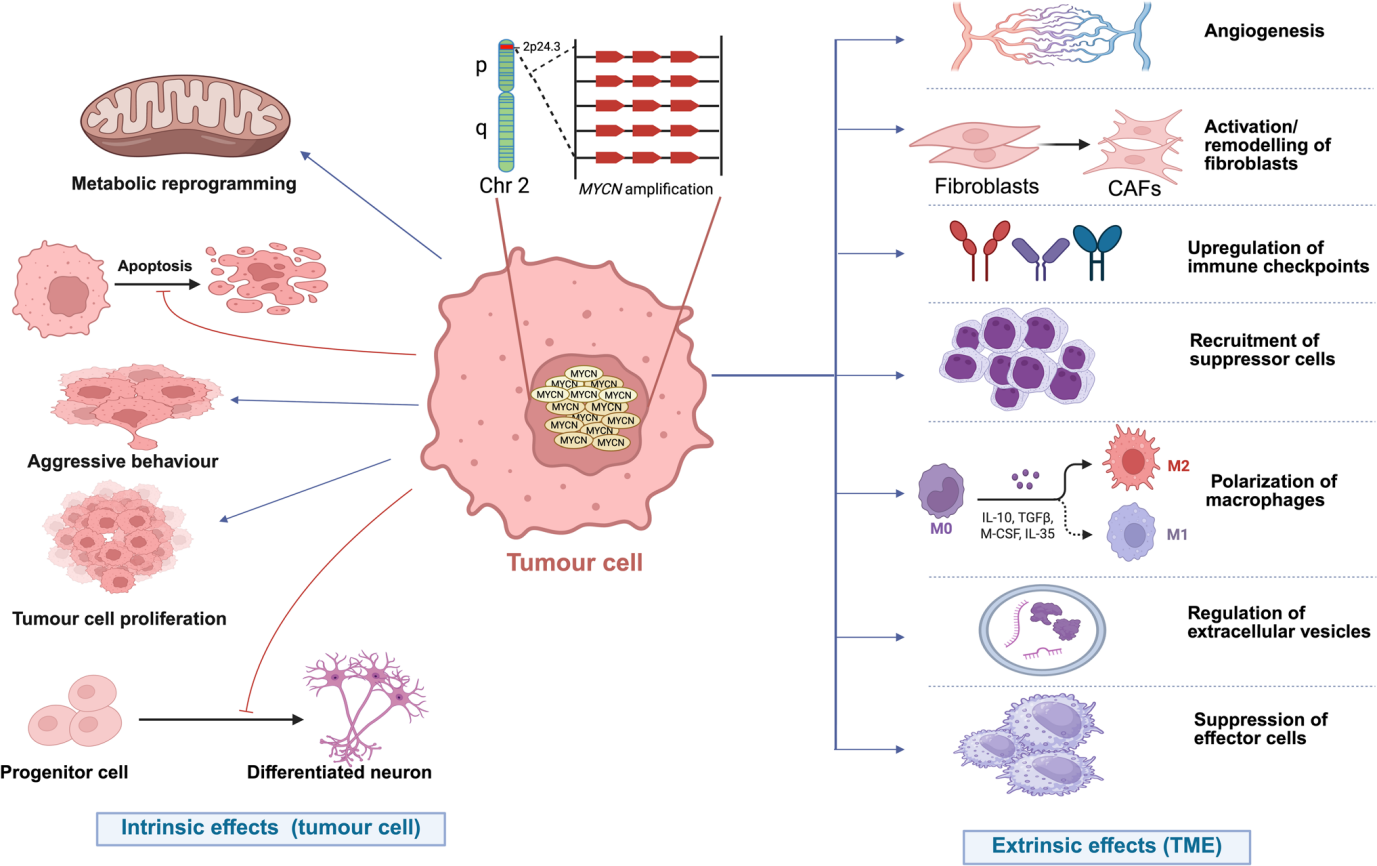
*MYCN***-**driven remodelling of NB tumour biology and the TME. Schematic overview of the proposed tumour-intrinsic and microenvironmental effects of *MYCN* amplification in NB. Tumour-intrinsic effects (left) metabolic reprogramming, evasion of apoptosis, include enhanced proliferation, and impaired neuronal differentiation collectively driving an aggressive tumour phenotype. In parallel, *MYCN*-associated extrinsic effects (right) extend to the TME, where they may promote angiogenesis, activation/remodelling of fibroblasts, upregulation of immune-checkpoints, recruitment or expansion of immunosuppressive cell populations, macrophage polarisation towards pro-tumourigenic states, altered extracellular vesicle-mediated communication and suppression of effector cells. Together, these intrinsic and extrinsic programmes contribute to ECM remodelling, immune exclusion and the establishment of a tumour-permissive, therapy-resistant niche. The red lines indicate negative impacts of *MYCN* (i.e. inhibition) of that cellular process. The inset highlights *MYCN* amplification at chromosome 2p24.3, illustrating increased *MYCN* copy number as a central genomic driver of aggressive NB biology

Collectively, current data support an association between *MYCN* amplification and reduced immune infiltration, altered NK-cell recognition, and skewed myeloid phenotypes. Together, these findings support a model in which *MYCN* amplification coordinates metabolic rewiring and immune suppression to reshape the NB TME (Fig. 1). However, causal mechanisms linking *MYCN* activity to immune exclusion or suppression remain incompletely defined. Future studies integrating *MYCN* status with multiplex imaging, spatial transcriptomics, and functional perturbation models will be essential to delineate how *MYCN* orchestrates immune remodelling within the NB TME.

Beyond immune cell–intrinsic alterations, the immunosuppressive phenotype of *MYCN*-amplified NB is reinforced by stromal remodelling, with CAFs serving as key architects of ECM composition, cytokine signalling, and metabolic crosstalk.

## CAFs and the TME

### Origin and activation of CAFs

Fibroblasts are spindle-shaped mesenchymal cells responsible for maintaining connective tissue integrity through ECM production. In cancer, these cells undergo activation into CAFs, acquiring proliferative, migratory, and secretory phenotypes reminiscent of wound healing and tissue repair programs [158–160].

CAFs arise from multiple cellular sources. While resident tissue fibroblasts represent a principal origin, additional contributors include pericytes and smooth muscle cells *via* transdifferentiation, epithelial cells though epithelial-mesenchymal transition (EMT), endothelial cells *via* endothelial-mesenchymal transition (EndMT), and bone marrow–derived mesenchymal stromal cells (MSCs) recruited to the tumour site [159, 161]. The diversity of cellular origins contributes to the marked heterogeneity observed within CAF populations.

**Fig. 2:**
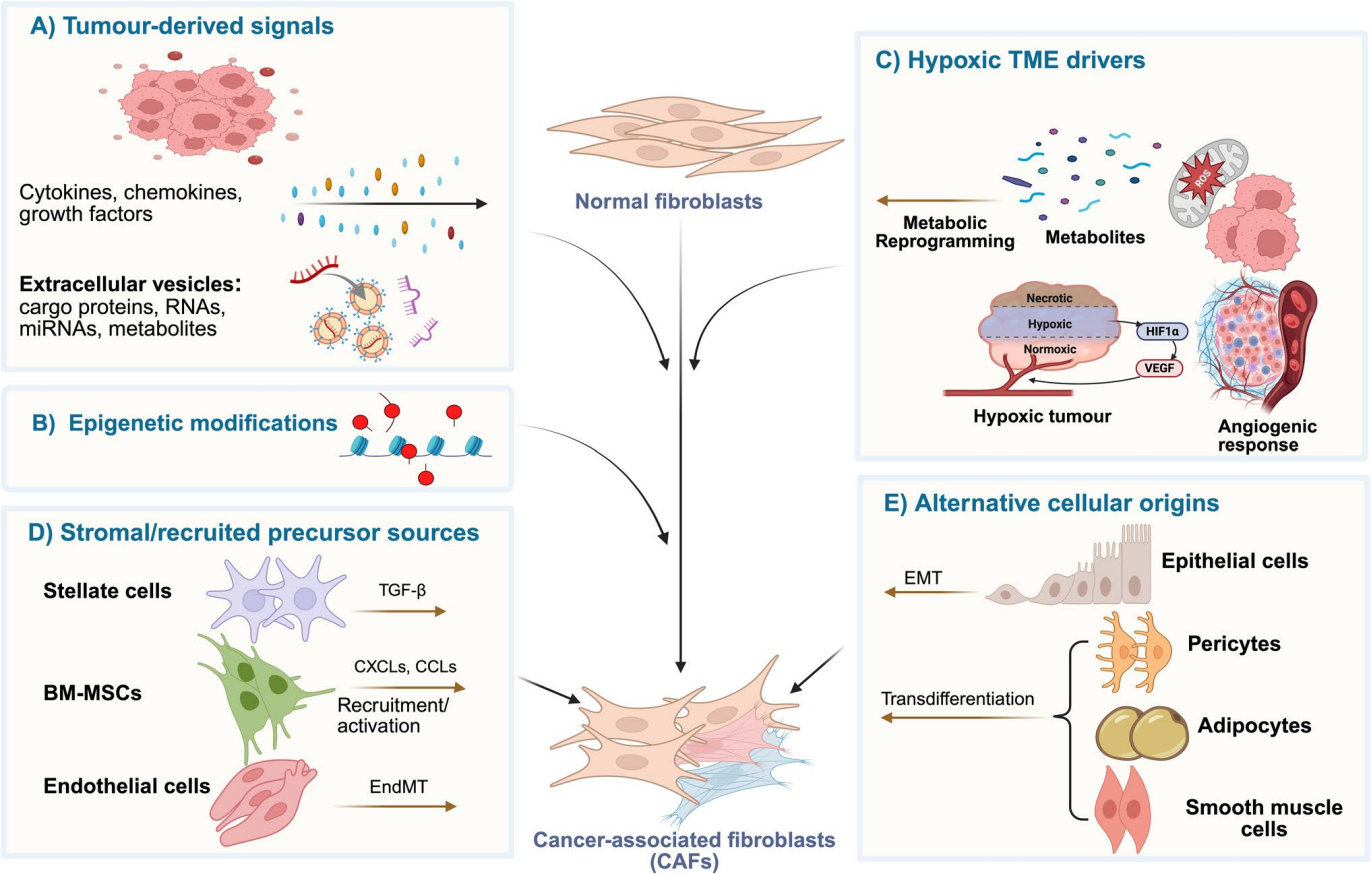
Cellular origins of CAFs and mechanisms of activation. **A** Resident normal fibroblasts can be converted into CAFs through tumour-derived signals, including cytokines, chemokines, growth factors and extracellular vesicles. **B** Epigenetic modifications also contribute to fibroblast activation by altering chromatin-associated regulatory programmes and sustaining CAF-associated phenotypes. **C** Hypoxic regions of the TME further promote CAF activation through hypoxia-induced signalling, ROS, altered metabolite availability, angiogenic responses and metabolic reprogramming. **D** In addition to resident fibroblasts, CAFs may originate from stromal or recruited precursor populations, including stellate cells activated by TGF-β, BM-MSCs recruited and activated by chemokines such as CXCLs and CCLs, and endothelial cells undergoing EndMT. **E** Alternative cellular sources may also contribute to the CAF pool, including epithelial cells through EMT, and pericytes, adipocytes and smooth muscle cells through trans-differentiation. The different CAF colours are used to represent heterogeneity within the CAF compartment. ***Abbreviations:*** BM-MSCs, bone marrow-derived mesenchymal stromal cells; CAFs, cancer-associated fibroblasts; EMT, epithelial–mesenchymal transition; EndMT, endothelial–mesenchymal transition; ROS, reactive oxygen species; TME, tumour microenvironment

Compared with normal fibroblasts, CAFs exhibit enhanced ECM deposition (e.g., collagen, fibronectin), secrete a broad spectrum of cytokines and chemokines, and actively modulate tumour cell survival, invasion, immune regulation, and therapeutic resistance [162]. The principal mechanisms underlying fibroblast activation and CAF diversification are summarized in Fig. 2.

### CAF signalling and communication with tumour and immune cells

CAFs engage in extensive bidirectional crosstalk with tumour cells, shaping tumour growth, metabolic adaptation, and therapeutic resistance. This communication is mediated through soluble factors, extracellular vesicles, metabolic intermediates, and ECM remodelling [163]. Growth factor signalling represents a major axis of tumour–CAF interaction. For example, CAF-derived fibroblast growth factors (FGFs) have been shown to stabilize MYC protein levels through activation of AKT and inhibition of GSK-3β, thereby enhancing tumour cell proliferation [164]. Similarly, activation of the IGF/IGF-1R pathway can promote oncogenic MYC signalling while simultaneously driving fibroblast activation, reinforcing a feed-forward tumour–stroma loop [165]. These findings illustrate how CAF-secreted factors can sustain oncogenic transcriptional programs in cancer cells.

Metabolic coupling constitutes another critical component of tumour–CAF communication. Under hypoxic conditions, CAFs and tumour cells engage in metabolic symbiosis mediated by lactate dehydrogenases (LDHs) and monocarboxylate transporters (MCTs), allowing dynamic switching between glycolysis and oxidative phosphorylation [166, 167]. CAF-derived lactate can fuel oxidative metabolism in tumour cells, while tumour-derived metabolites can reciprocally reprogram CAF metabolism [168]. Beyond glycolysis, lipid metabolic reprogramming in CAFs—including upregulation of fatty acid synthesis pathways—has been implicated in promoting tumour migration and aggressiveness in several solid tumours [169].

In addition to tumour cell interactions, CAFs exert profound effects on immune regulation. Through secretion of cytokines, chemokines, and ECM components, CAFs can modulate immune cell recruitment, polarization, and effector function, often contributing to an immunosuppressive microenvironment [170]. In multiple tumour types, selective targeting of specific CAF populations has been shown to enhance anti-tumour immunity and improve responses to immunotherapy [171–175].

Collectively, these studies demonstrate that CAFs function as central signalling hubs within the TME, integrating growth factor, metabolic, and immune-modulatory pathways to sustain tumour progression. Whether similar tumour–CAF signalling networks operate in NB, particularly in the context of *MYCN*-driven biology, remains an important area of investigation.

### Functional and phenotypic heterogeneity of CAFs

CAFs represent a highly heterogeneous population of mesenchymal cells characterized by diverse phenotypic and functional states [21, 176, 177]. Historically, CAFs have been identified using markers such as fibroblast-specific protein-1 (FSP-1), α-smooth muscle actin (α-SMA), fibroblast activation protein-α (FAP-α), platelet-derived growth factor receptors (PDGFRα/β), and integrins [178, 179]. However, none of these markers are exclusive to CAFs, as they are also expressed by other stromal and immune cell populations within the TME [159, 180, 181]. Consequently, no single marker adequately defines the CAF compartment, and combinational marker strategies are required to capture its full complexity [182].

Advances in single-cell transcriptomics and spatial profiling have further revealed substantial inter- and intra-tumour heterogeneity of CAFs across adult malignancies [183–188]. Distinct CAF subsets with inflammatory, myofibroblastic, antigen-presenting, metabolic, or tumour-restraining features have been described, underscoring the functional specialization within the stromal compartment. Selected examples of CAF subtypes and associated biomarkers identified in adult cancers are summarised in Table 2.

**Table 2:** CAF heterogeneity and selected biomarkers in adult cancers

Cancer type(s)	Technology/methods	CAF subtypes and key biomarkers	Functional and/or spatial insights	References
Breast cancer (primary cohort) and additional adult cancers (colon, HNSCC, PDAC, NSCLC)	• scRNA-seq • Imaging Mass cytometry (proteomics)	Nine CAF subtypes identified: ***mCAFs (matrix/contractile):*** MMP11, COL1A1, COL1A2 ***iCAFs (inflammatory):*** PLA2G2A, CD34, IL6 ***apCAFs (antigen-presenting):*** MHC-II molecules, CD74 ***vCAFs (vascular-associated):*** NOTCH3, COL18A1, COL4A1 ***tCAFs (tumour-associated):*** CAIX, PDPN infCAFs (interferon-responsive): IL32, IDO1 ***rCAFs (reticular):*** CCL21, CCL19 ***dCAFs (dividing/proliferative):*** TUBA1B, MKI67 ***hsptCAFs (heat-shock):*** HSPH1, HSP90AA1	mCAFs: ECM remodelling iCAFs: inflammatory signalling apCAFs: antigen presentation vCAFs: angiogenesis tCAFs: tumour growth/metastasis rCAFs: naïve T-cell homing dCAFs: proliferation hsptCAFs: stress response	[186]
Lung and breast cancers	• mRNA microarray • Flow cytometry • Immunohistochemistry	***CD10⁺/GPR77⁺ CAF subset***	Promotes cancer stemness and chemoresistance	[189]
PDAC (human cohorts)	• scRNA-seq • Bulk RNA-seq (deconvolution)	Three major CAF subsets: ***myCAFs:*** ACTA2 (α-SMA), S100A4, MYL9 ***iCAFs:*** PDGFRα, IL6, CXCL12 ***apCAFs:*** CD74, HLA-DRA, HLA-DRB1	iCAFs: glycolysis-biased and inflammatory myCAFs: OXPHOS/TCA-cycle enriched apCAFs: antigen processing and presentation	[187]
PDAC (human and KPC mouse model)	• scRNA-seq	***myCAFs:*** ACTA2, TAGLN, MMP11, MYL9, POSTN ***iCAFs:*** PDGFRα, CXCL12, CCL2, CFD ***apCAFs (mouse):*** H2-Aa, H2-Ab1, Cd74	myCAFs: adjacent to tumour cells iCAFs: localised to desmoplastic stroma CAF lineage influences tumour immunity and progression	[190]
Skin cancers (BCC, SCC, melanoma)	• Smart-seq2 scRNA-seq • Spatial transcriptomics	Three major CAF groups: ***RGS5⁺ CAFs (pericyte-like):*** RGS5, ACTA2, PDGFRB, TAGLN ***mCAFs:*** COL1A1, COL1A2, COL3A1, POSTN, TNC ***iCAFs*** (immunomodulatory): MMP1, MMP3, IDO1, IL6, CXCL8	mCAFs: ECM deposition; may restrict T-cell infiltration iCAFs: cytokine/chemokine secretion Subtype abundance shifts with tumour malignancy	[188]
Prostate cancer	• scRNA-seq • Immunofluorescence	***FerroCAFs (iron-loaded CAFs):*** CCL2, CSF1, CXCL1	Recruit and sustain immunosuppressive myeloid cells	[191]

*apCAFs*, Antigen-presenting CAFs; *ECM*, Extracellular matrix; *dCAFs*, Dividing/proliferative CAFs; *iCAFs*, Inflammatory CAFs; *mCAFs*, Matrix-producing CAFs; *myCAFs*, Myofibroblastic CAFs; *vCAFs*, Vascular-associated CAFs; *rCAFs*, Reticular CAFs; *tCAFs*, Tumour-associated CAFs; *infCAFs*, Interferon-responsive CAFs; *HSP-high CAFs*, Heat-shock protein–enriched CAFs; *PDAC*, Pancreatic ductal adenocarcinoma; *NSCLC*, Non-small cell lung cancer; *scRNA-seq*, Single-cell RNA sequencing; KPC, Kras^G12D^/+; Trp53^R172H/+^; Pdx1-Cre

Collectively, these findings highlight that CAFs comprise multiple transcriptionally and functionally distinct states rather than a uniform stromal entity. This conceptual framework provides a basis for interrogating CAF identity and diversity in paediatric tumours such as NB.

Beyond phenotypic diversity, CAF subsets display marked functional heterogeneity, and their net impact on tumour progression appears highly context dependent. While CAFs are frequently described as tumour-promoting facilitating immune suppression, tumour growth, invasion, and metastasis, emerging evidence indicates that certain CAF populations may exert tumour-restraining or immune-supportive functions [192–194].

Studies in pancreatic cancer have provided some of the clearest demonstrations of this functional dichotomy. For example, CD105-defined CAF subsets display opposing roles, with CD105⁺ CAFs promoting tumour growth and CD105⁻ CAFs exhibiting tumour-suppressive activity [195]. Similarly, depletion of α-SMA⁺ CAFs accelerated pancreatic tumour progression, resulting in increased hypoxia, dedifferentiation, and stemness [196], highlighting the risks of indiscriminate stromal targeting. Distinct inflammatory (iCAF) and myofibroblastic (myCAF) states have also been described, with iCAFs typically characterized by high IL-6 production and cytokine secretion, and myCAFs associated with ECM deposition [197–199]. However, the functional consequences of these states vary across tumour types; for instance, iCAF signatures have been linked to favourable prognosis in certain contexts such as non-small cell lung cancer [200].

Collectively, these findings underscore that CAF composition, rather than total CAF abundance alone, influences tumour immunity, therapeutic response, and patient outcome across adult malignancies [187, 200–202] Selected associations between CAF subsets, immunomodulatory functions, and clinical prognosis are summarized in Table 3. These observations reinforce the need to define CAF diversity in a tumour-specific manner and caution against generalized assumptions regarding CAF function.

**Table 3:** CAF composition and functions in immunomodulation and patient prognosis

Cancer type	Technologies employed	Key findings	Reference
NSCLC	Imaging Mass Cytometry	Presence of tumour-associated CAFs (tCAFs) correlated with poor prognosis. Inflammatory CAFs (iCAFs) and interferon-responsive CAFs (infCAFs) were associated with an inflamed TME and improved survival. High-density matrix CAFs (mCAFs) were linked to reduced immune infiltration and inferior patient outcomes	[200]
NSCLC	scRNA-seq; Flow cytometry; Multiplex immunofluorescence	Five broad CAF subsets (CAFS1–CAFS5) identified. The CAFS1 subset, characterised by strong PDPN and FAP expression, was associated with poor patient survival	[201]
Gastric cancer	Immunohistochemistry	CD10⁺, FAP⁺ and GPR77⁺ CAFs were associated with chemoresistance and poor prognosis	[202]
PDAC	scRNA-seq; Bulk RNA-seq; Immunohistochemistry	Inflammatory CAFs (iCAFs) were associated with a favourable prognostic phenotype, whereas myofibroblastic CAFs (myCAFs) correlated with poor outcome	[187]
High-grade serous ovarian cancer (HGSOC)	scRNA-seq; Spatial transcriptomics	*NNMT* activity in CAFs causes H3K27me3 hypomethylation, which in turn upregulates complement secretion and recruits immunosuppressive MDSCs. *NNMT* blockade in CAFs restores the efficacy of immune checkpoint blockade, limits CAF-mediated MDSC recruitment and reinvigorates CD8⁺ T cells	[203]

*PDAC*, Pancreatic ductal adenocarcinoma; *NSCLC*, Non-small cell lung cancer; *HGSOC*, High-grade serous ovarian cancer; *MDSCs*, Myeloid-derived suppressor cells; *tCAFs*, Tumour-associated CAFs; *mCAFs*, Matrix-producing CAFs; *infCAFs*, Interferon-responsive CAFs; *IHC*, Immunohistochemistry; *IF*, Immunofluorescence

### CAFs in the context of the NB TME

#### CAF/MSCs signalling and crosstalk in NB

In contrast to the extensive characterization of CAF subsets in adult malignancies, the identity and functional contribution of CAFs in NB remain incompletely defined. Nevertheless, emerging evidence suggests that stromal fibroblast-like populations participate in coordinated immunosuppressive and tumour-promoting networks within the NB TME.

Early immunohistochemical studies demonstrated increased α-SMA⁺, non-pericytic (h-caldesmon⁻) CAF-like areas in *MYCN*-amplified tumours compared to non-amplified counterparts, with CAF abundance correlating with high-risk disease and bone marrow metastasis [204]. Notably, TAMs were frequently localized adjacent to CAF-rich regions, and their density increased in parallel with CAF grade, suggesting the presence of structured stromal–immune niches. *In vitro*, NB-conditioned medium was sufficient to polarize peripheral blood mononuclear cell–derived macrophages toward a TAM-like phenotype and to drive bone marrow–derived mesenchymal stromal cells (BM-MSCs) toward a CAF-like state, supporting reciprocal tumour–stroma programming.

Subsequent studies further highlighted coordinated CAF–TAM interactions in NB. CAF/MSC populations enhanced tumour cell proliferation, survival, and chemotherapy resistance *in vitro* and promoted tumour engraftment *in vivo* [205]. Correlative analyses revealed positive associations between CAF markers (e.g., FAP, FSP-1) and TAM markers (e.g., CD163), reinforcing the concept of CAF–TAM co-enrichment in high-risk disease. Mechanistically, CAF–TAM crosstalk has been linked to activation of the TGF-β/IL-6 axis, sustaining macrophage survival and polarization while dampening NK-cell activity [206]. Complementary findings indicate that NB-associated MSCs can directly suppress NK-cell function through expression of immunomodulatory molecules [207].

**Fig. 3:**
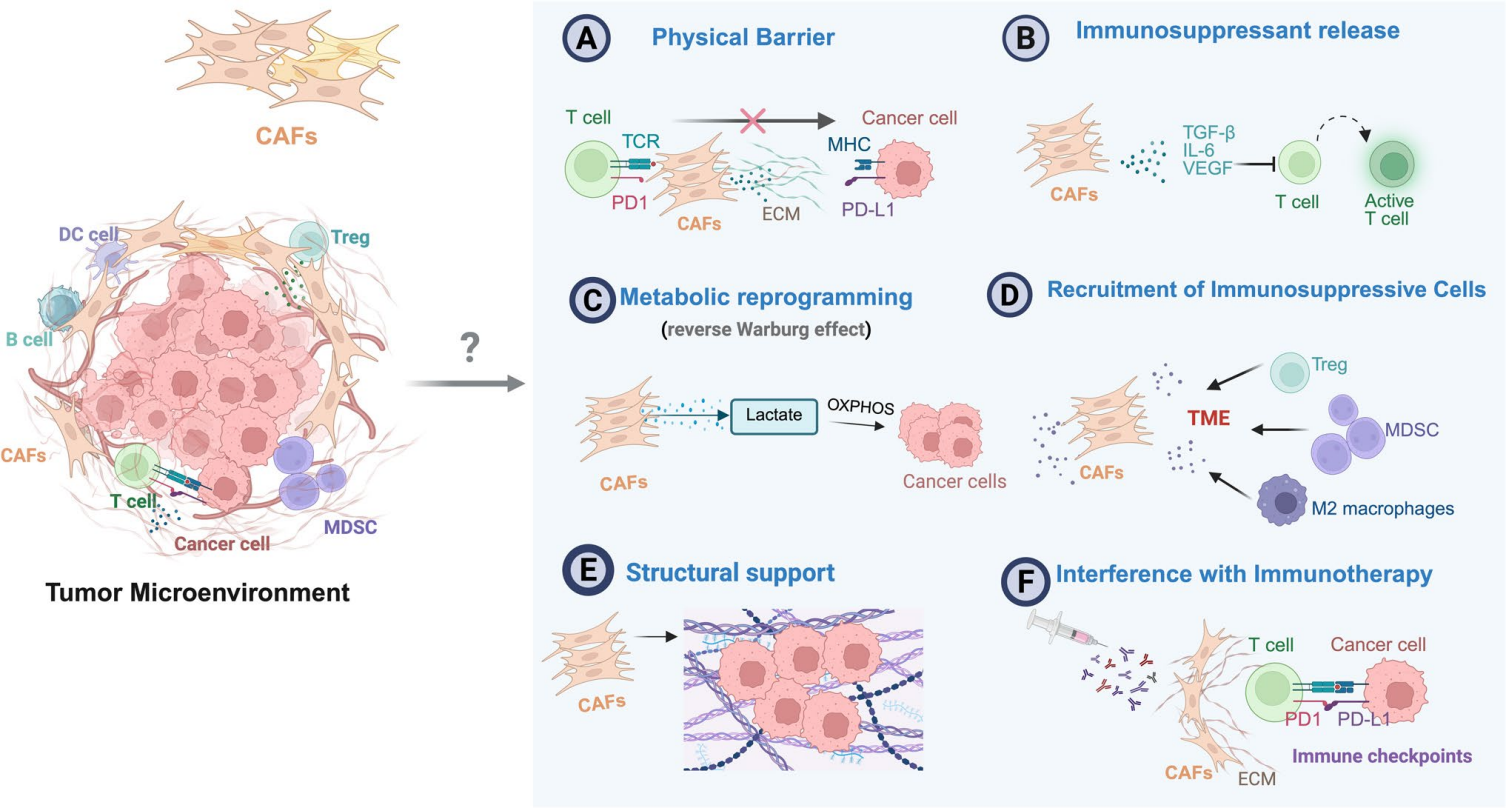
The potential roles of CAFs in reshaping the TME. **A** Physical barrier: CAFs and CAF-generated ECM forms a dense stroma that limits T-cell–tumour contact and effective TCR–MHC engagement. **B** Immunosuppressant release: CAFs secrete TGF-β, IL-6 and other factors that blunt cytotoxic T-cell activity and promote regulatory phenotypes. **C** Metabolic reprogramming: metabolically reprogrammed CAFs can release lactate, supporting tumour OXPHOS and contributing to the “reverse Warburg effect.” **D** Recruitment of immunosuppressive cells: CAF-derived chemokines attract and polarise Tregs, M2 macrophages and MDSCs. **E** Structural support: ECM deposition/remodelling provides mechanical support, increases stiffness and creates invasion tracks. **F** Interference with immunotherapy: CAF cues and ECM impede drug and cell infiltration and can modulate immune checkpoints (e.g., PD-1/PD-L1), reducing response to therapy. ***Abbreviations:*** CAF(s), cancer-associated fibroblast(s); TME, tumour microenvironment; ECM, extracellular matrix; TCR, T-cell receptor; MHC, major histocompatibility complex; PD-1/PD-L1, programmed cell death protein-1/ligand-1; Treg(s), regulatory T cell(s); MDSC(s), myeloid-derived suppressor cell(s); OXPHOS, oxidative phosphorylation

Collectively, these studies suggest that NB tumours harbor stromal–immune niches characterized by CAF/MSC–TAM cooperation and cytokine-driven immunosuppression. However, current evidence is largely based on conventional marker-defined populations and *in vitro* co-culture systems. The transcriptional heterogeneity, spatial organization, and lineage origins of CAF subsets in primary NB tumours remain poorly resolved. A schematic summary of proposed CAF-mediated mechanisms in NB is presented in Fig. 3.

#### Advances in CAF diversity and clinical correlates in NB

Recent single-cell transcriptomic studies have begun to delineate CAF heterogeneity in NB. Using scRNA-seq, Costa et al. identified a CAF cluster in TH-MYCN mouse tumours characterized by Fn1 expression, which further resolved into two subsets: an immunomodulatory CAF-S1 population enriched for chemokines such as *Ccl2*, *Cxcl1*, and *Cxcl12*, and a CAF-S4 subset with features suggestive of pro-metastatic and perivascular activity. Parallel analysis of human NB biopsies revealed analogous COL1A1⁺ CAF populations, with CAF-S1 similarly expressing *Cxcl12* and *Ccl2*, supporting cross-species conservation of immunomodulatory CAF states [151]. These findings provide some of the first evidence for transcriptionally distinct CAF subsets in NB and suggest functional specialization within the stromal compartment.

Building on single-cell datasets, several groups have developed CAF-related prognostic signatures by integrating scRNA-seq–derived marker genes with bulk transcriptomic cohorts. Cao et al. identified 253 CAF-associated genes from eight NB tumours and constructed an eight-gene risk model that stratified patients into prognostic groups across independent datasets. The derived CAF risk score correlated with immune infiltration patterns and predicted therapeutic response [208]. Similarly, other studies have proposed CAF-based prognostic models to predict survival and immunotherapy sensitivity in NB [209, 210].

While these integrative approaches underscore the potential clinical relevance of CAF-associated transcriptional programs, several limitations warrant consideration. Many proposed CAF markers and risk models are derived primarily from computational intersection of bulk and single-cell datasets, without definitive validation of fibroblast-specific expression *in situ*. Subtype definitions are often based on transcriptomic scoring rather than orthogonal validation using spatial transcriptomics, multiplex imaging, or protein-level analyses. Moreover, benchmarking against established CAF states described in adult cancers remains inconsistent. As a result, the precise identity, lineage origin, and functional roles of proposed CAF subsets in NB remain incompletely resolved.

Taken together, emerging single-cell and integrative analyses suggest that CAF diversity in NB may parallel certain features described in adult malignancies, particularly immunomodulatory and perivascular-like subsets. However, a consensus framework for defining CAF states in NB has yet to be established, and further studies integrating spatially resolved and functional validation approaches are required.

#### Gaps in understanding CAF heterogeneity in the NB TME

Despite growing interest in stromal biology, the identity, diversity, and functional significance of CAFs in NB remain incompletely defined. Most existing NB studies have focused on MSC–CAF interactions *in vitro* or have characterized CAFs using single-marker approaches (e.g., α-SMA or FAP), thereby treating CAFs as a homogeneous population [204–209]. Comprehensive *in vivo* characterization of CAF subsets using integrative single-cell, spatial, and functional platforms is lacking.

A major unresolved challenge is distinguishing tumour-intrinsic mesenchymal (MES) states from bona fide stromal fibroblast populations. Given the substantial overlap in biomarkers between MES tumour programs and CAF/MSC signatures, unbiased identification of CAF subsets in primary NB tumours remains technically and conceptually complex [10, 11, 15, 17]. Rigorous lineage tracing, spatial transcriptomics, and multiplex imaging approaches will be required to clarify stromal *versus* tumour-derived mesenchymal states.

Furthermore, CAF heterogeneity has not been systematically examined in relation to molecular and clinical covariates such as *MYCN* amplification, *MYCN* expression levels, treatment exposure, or disease stage. Given the central role of *MYCN* in metabolic rewiring and immune modulation, it is plausible that *MYCN* status influences CAF composition, phenotype, and metabolic crosstalk within the TME. However, such stratified analyses remain largely unexplored.

Finally, while adult cancer studies have established links between specific CAF subsets and therapeutic response, metastasis, and immune regulation, it is uncertain whether analogous CAF states and functions exist in paediatric tumours such as NB. Whether CAF composition influences response to chemotherapy, anti-GD2 immunotherapy, or emerging targeted agents in NB remains an open question.

Addressing these gaps will require integrated multi-omic, spatially resolved, and longitudinal analyses to define CAF lineage, functional specialization, and tumour–stroma interactions across disease stages and key outstanding questions are outlined in Table 4.

**Table 4:** Key knowledge gaps in CAF biology in NB and proposed research directions

Potential knowledge gap	Current knowledge at a glance	Future directions
Therapeutic Targets	Current treatments primarily target tumour cells (chemotherapy) and immune cells (immunotherapy). Stromal populations, including CAFs, remain largely untargeted. No established CAF-directed therapies in NB	Develop stromal-targeted strategies informed by mechanistic studies of CAF biology. Identify actionable CAF-specific pathways (e.g., metabolic or ECM-associated) to overcome resistance and relapse
Phenotypic Characterization (Biomarkers)	Tumour cells: PanCK, EpCAM, MYCN/N-MYC, PHOX2B Immune cells: CD45 CAFs: lack of specific, consistent markers; significant heterogeneity across cancers and limited characterisation in NB	Use multi-marker panels derived from scRNA-seq to define CAF subsets. Integrate single-cell and spatial multi-omic approaches (scRNA-seq, imaging mass cytometry, multiplex immunohistochemistry) to accurately resolve stromal complexity
CAF Heterogeneity and Function	Adult cancers define functional CAF states [186, 187]: ECM-remodelling (myCAFs/mCAFs), inflammatory (iCAFs), vascular (vCAFs), antigen-presenting (apCAFs). Conflicting roles reported (tumour-promoting *vs* tumour-suppressive). Limited data in NB	Perform comprehensive profiling of CAF subsets in NB using single-cell and spatial multi-omics. Combine phenotypic characterisation with functional validation in physiologically relevant models
CAFs Classification Systems	No universal CAF classification system, particularly in paediatric tumours. Single markers insufficient to define CAF identity or subsets	Develop integrative classification frameworks using multi-marker signatures derived from transcriptomic data. Standardise CAF subtype annotation across studies
Cellular Communication	Tumour–CAF and CAF–immune metabolic crosstalk suggested but incompletely characterised in NB. Mechanistic pathways remain poorly defined	Dissect CAF–tumour and CAF–immune interactions using 3D co-culture systems, conditioned media, and transwell assays. Apply metabolic and proteomic profiling (e.g., Seahorse analysis, mass spectrometry) and spatial imaging to define signalling axes
Roles in Disease Progression	Limited evidence linking CAF composition to prognosis or therapy response in NB	Correlate CAF subtype abundance and gene signatures with survival, treatment response, and MYCN status by integrating single-cell and bulk transcriptomic datasets
Technologies and Research Models	NB research has predominantly used conventional IHC (DAB) and limited-channel IF. Most functional studies rely on 2D monolayer cultures and simplified co-culture systems	Implement advanced multiplex spatial imaging platforms and physiologically relevant models (3D spheroids, patient-derived organoids) to capture TME heterogeneity and dynamic interactions

## Conclusion and future perspectives

Neuroblastoma remains a highly heterogeneous disease in which *MYCN* amplification drives aggressive tumour behaviour, metabolic rewiring, and poor clinical outcome. While significant progress has been made in understanding tumour-intrinsic *MYCN* biology, this review highlights an emerging paradigm in which *MYCN* functions as a central orchestrator of TME remodelling. In particular, CAFs are increasingly recognised as key mediators of extracellular matrix organisation, immune modulation, and metabolic crosstalk, yet their identity, heterogeneity, and functional roles in neuroblastoma remain incompletely defined.

A major unresolved challenge is the distinction between tumour-intrinsic mesenchymal programs and bona fide stromal populations, which has complicated interpretation of single-cell datasets and may have obscured the true contribution of CAFs to tumour progression and therapeutic response. Addressing this ambiguity, alongside defining CAF functional states and lineage relationships, will be essential for advancing our understanding of stromal biology in neuroblastoma.

Importantly, the concepts outlined in this review extend beyond neuroblastoma. *MYCN* and *MYC*-driven metabolic and microenvironmental reprogramming are increasingly recognised across multiple malignancies, including medulloblastoma, small cell lung cancer, and other *MYC*-family–driven tumours. In this context, neuroblastoma provides a valuable model system to interrogate how oncogenic transcription factors reshape tumour ecosystems through coordinated effects on metabolism, stromal compartments, and immune regulation.

Future research should prioritise integrative, spatially resolved, and functionally validated approaches to define CAF heterogeneity and tumour–stroma interactions across disease states and treatment contexts. These efforts will be critical for identifying actionable stromal vulnerabilities and for developing combination strategies that target both tumour cells and their supportive niche.

Ultimately, resolving *MYCN*-driven tumour–microenvironment interactions may enable more effective and less toxic therapeutic strategies, with implications not only for neuroblastoma but for a broader class of MYC-driven cancers.

## Data Availability

No datasets were generated or analysed during the current study.

## Abbreviations

ACC: Acetyl-CoA carboxylase
ADCC: Antibody-dependent cellular cytotoxicity
ALDH18A1: Aldehyde dehydrogenase 18 family member A1
ALK: Anaplastic lymphoma kinase
ALT: Alternative lengthening of telomeres
apCAF: Antigen-presenting cancer-associated fibroblast
ATP: Adenosine triphosphate
ATRX: Alpha-thalassaemia/mental retardation syndrome X-linked
CAF: Cancer-associated fibroblast
CD: Cluster of differentiation
COL1A1: Collagen type I alpha 1 chain
CXCL: C-X-C motif chemokine ligand
DFMO: Difluoromethylornithine
ECM: Extracellular matrix
EMT: Epithelial–mesenchymal transition
EndMT: Endothelial–mesenchymal transition
FAP: Fibroblast activation protein
FASN: Fatty acid synthase
GD2: Disialoganglioside
GSEA: Gene set enrichment analysis
HGSOC: High-grade serous ovarian cancer
HIF: Hypoxia-inducible factor
HK2: Hexokinase 2
IF: Immunofluorescence
IHC: Immunohistochemistry
iCAF: Inflammatory cancer-associated fibroblast
IL: Interleukin
IMC: Imaging mass cytometry
KAT2A: Lysine acetyltransferase 2A
KPC: Kras^G12D/ + ; Trp53^R172H/ + ; Cre
LDH: Lactate dehydrogenase
mCAF: Matrix cancer-associated fibroblast
MSC: Mesenchymal stromal cell
MDSC: Myeloid-derived suppressor cell
MYCN: v-myc avian myelocytomatosis viral related oncogene neuroblastoma derived
NB: Neuroblastoma
NK: Natural killer
NSCLC: Non-small cell lung cancer
NSCLC: Non-small cell lung cancer
ODC1: Ornithine decarboxylase 1
OXPHOS: Oxidative phosphorylation
PBMC: Peripheral blood mononuclear cell
PDAC: Pancreatic ductal adenocarcinoma
PDGFR: Platelet-derived growth factor receptor
PHOX2B: Paired-like homeobox 2b
POSTN: Periostin
scRNA-seq: Single-cell RNA sequencing
SOX10: SRY-box transcription factor 10
TAM: Tumour-associated macrophage
TCA: Tricarboxylic acid
*TERT*: Telomerase reverse transcriptase
TGF-β: Transforming growth factor beta
TME: Tumour microenvironment
